# A muscle-centered hierarchical breakdown underlies flight loss during silkworm domestication

**DOI:** 10.1016/j.isci.2026.114846

**Published:** 2026-01-29

**Authors:** Rongpeng Liu, Chen Zhao, Jie Hu, Yongbing Ba, Yiting Ran, Yuanyuan Mu, Yiyun Tang, Yan Ma, Zhiming Zhang, Kaiqi Guo, Keshu Dong, Xiao Li, Yumeng Zhu, Wei Tan, Hanfu Xu

**Affiliations:** 1 State Key Laboratory of Resource Insects, College of Sericulture, Textile and Biomass Sciences, Southwest University, Chongqing 400715, China; 2 Shanghai OE Biotech. Co., Ltd., Shanghai 201212, China; 3 Institute for Silk and Related Biomaterials Research, Chongqing Academy of Animal Sciences, Chongqing 402460, China

**Keywords:** Entomology, Molecular biology, Evolutionary biology, Transcriptomics

## Abstract

Recurrent loss of complex traits such as insect flight offers insights into evolutionary regression, as seen in *Bombyx mori* domestication from its flight-capable ancestor *B. mandarina*. By integrating single-cell and spatial transcriptomics of developing flight organs in both *Bombyx* species, we reveal that flight loss occurs through a coordinated breakdown centered on flight muscle cells. This stems from disintegration of a multi-tiered genetic module with three interconnected components: failures in mitochondrial energy production (e.g., *COX3/ND1*), wing vein patterning (involving *Dally/CtBP*), and flight muscle specification (controlled by *Yki*). Knocking down key components in *B. mandarina* induced flightlessness, and perturbing the Hippo effector Yki in *B. mori* exacerbated wing defects, together highlighting the pathway’s dosage-sensitive nature. Notably, this muscle-centric module is conserved in the migratory pest *Helicoverpa armigera*, where its disruption similarly impaired flight. Our work establishes trait degeneration as a muscle-centered collapse and identifies a conserved target for precision pest control.

## Highlights

• Single-cell and spatial atlas reveals a muscle-centric basis for flight loss in *Lepidoptera*
• Flight degeneration follows hierarchical disintegration of a three-tiered genetic module
• Conserved flight module in *Helicoverpa armigera* informs precision pest control

## Graphical abstract

**Figure:**
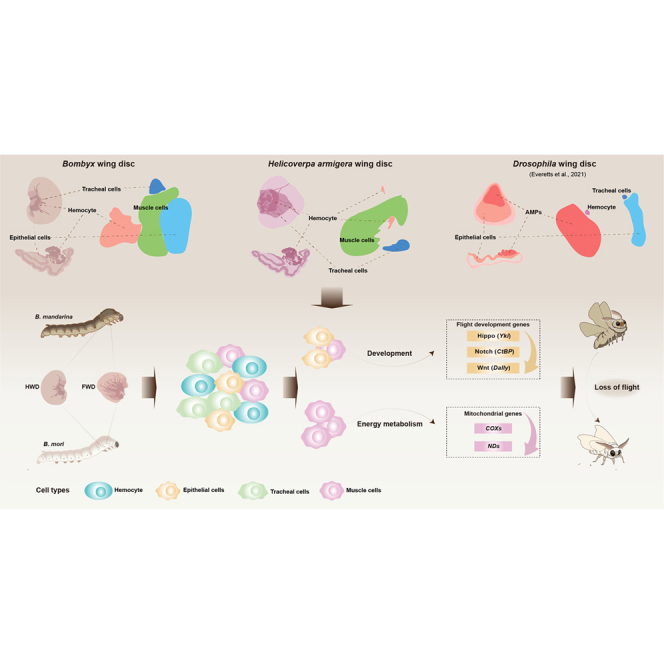

## Introduction

Flight capacity represents a landmark innovation in animal evolution, enabling insects to dominate terrestrial ecosystems through predator evasion, resource acquisition, and long-range dispersal.^1^ Paradoxically, evolutionary flight loss has emerged independently across numerous insect lineages, with lepidopterans exhibiting particularly frequent transitions between flight-capable and flight-deficient states.^2^^,^^3^^,^^4^ While molecular studies in dipterans such as *Drosophila melanogaster* have established fundamental principles of wing development and flight muscle specification,^5^^,^^6^^,^^7^ critical gaps persist in understanding how these systems degrade during evolutionary flight loss, a phenomenon with profound implications for both developmental biology and agricultural pest management.

**Figure 1:**
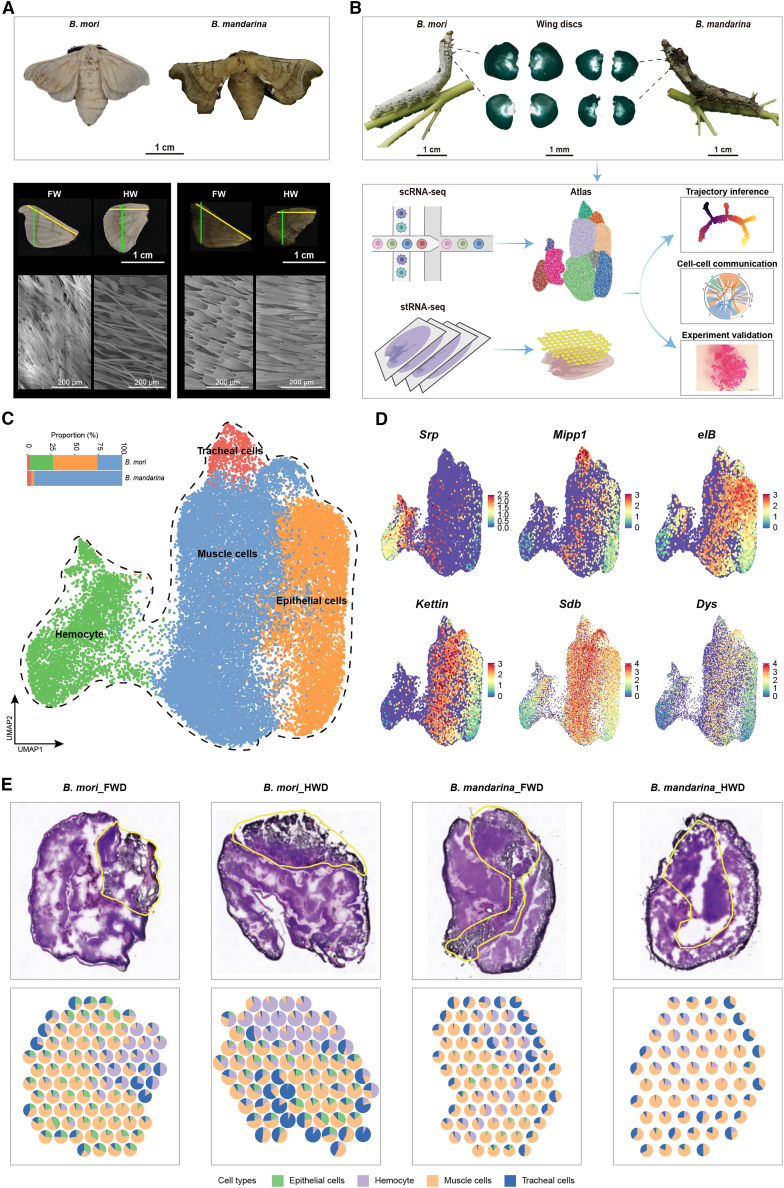
Single-cell transcriptomic atlas and spatial localization of silkworm flight organs (A) Adult wing morphology and scanning electron microscopy images of forewing (FW) and hindwing (HW) scales from *B. mori* and *B. mandarina*. Scale bars, 1 cm for adults and wings, 200 μm for enlarged images. (B) Schematic workflow. Wing discs from the second and third thoracic segments of fifth-instar larvae of *B. mori* and *B. mandarina* were dissected, dissociated, and subjected to single-cell RNA sequencing (scRNA-seq) or spatial transcriptomics (ST). Following quality control, the scRNA-seq and ST datasets were integrated for downstream analysis and experimental validation. Scale bars, 1 cm for larvae and 1 mm for wing discs. (C) Left: Bar charts showing the proportional abundance of each cell type in *B. mori* and *B. mandarina*. Right: UMAP visualization of the four annotated cell types in *B. mori* and *B. mandarina*. (D) Feature plots of representative marker genes used for cell type annotation in the wing discs of *B. mori* and *B. mandarina*. (E) Top: H&E staining of forewing discs (FWD) and hindwing discs (HWD) from *B. mori* and *B. mandarina*. Yellow dashed lines indicate hematopoietic organs. Bottom: spatial mapping of the four cell types via robust cell-type decomposition (RCTD) on ST data.

The domestic silkworm (*Bombyx mori*), a well-established lepidopteran model organism,^8^ presents a unique opportunity to investigate flight regression. Unlike its wild ancestor *Bombyx mandarina*—a robust flyer capable of long-distance dispersal—*B. mori* retains vestigial wings but has completely lost flight capacity following over 5,000 years of domestication.^9^^,^^10^^,^^11^ This recent evolutionary divergence, illustrated by the morphological comparison in Figures 1A and S1, provides an exceptional comparative system for pinpointing the cellular and molecular modifications driving functional degradation of flight organs.

Despite its significance, the lepidopteran flight apparatus remains poorly characterized compared to dipteran models, particularly regarding high-resolution cellular architectures, spatial gene expression patterns, and interspecies conservation of flight-related pathways.^12^^,^^13^^,^^14^ This knowledge gap stems from two key factors. First, traditional models like *Drosophila* exhibit fundamental anatomical divergences from Lepidoptera, including distinct wing-scale morphogenesis, flight muscle organization, and energy metabolism strategies.^15^^,^^16^ Second, although bulk transcriptomic studies in silkworms have implicated factors such as flight muscle degeneration and specific genes (e.g., *fln*, *COX*) in flight loss,^10^^,^^17^ they are unable to resolve the cellular heterogeneity within flight organs. This is a critical limitation, as flight capacity depends on coordinated interactions among diverse cell types, including epithelial cells (which form the wing structure), adult muscle precursors (responsible for flight muscles), and metabolic support cells.^18^^,^^19^ A high-resolution, cell-centric analysis is therefore essential to uncover the key molecular mechanisms driving flight loss in silkworms.

To address these challenges, we employed an integrated approach combining single-cell RNA sequencing (scRNA-seq) and spatial transcriptomics to compare flight organs of *B. mori* and *B. mandarina*. This strategy enabled us to systematically map the cellular and molecular landscapes of *lepidopteran* flight organs. Our analysis reveals that muscle cell dysfunction is the primary driver of flight loss and identifies a hierarchical, three-tiered genetic module whose disintegration underpins this functional decline. Through functional validation in both silkworms and the cotton pest *Helicoverpa armigera*, we demonstrate the causal role and evolutionary conservation of this module. By resolving the spatiotemporal basis of flight loss, this work advances our understanding of evolutionary trait regression and identifies conserved targets for pest management.

## Results

### Single-cell transcriptome atlas of flight organs in silkworms

We constructed a single-cell transcriptome atlas of the forewing discs (FWD) and hindwing discs (HWD) from fifth-instar larvae of *B. mori* and its wild ancestor *B. mandarina* to systematically characterize the cellular landscape of silkworm flight organs (Figure 1B). After strict quality control, 27,656 high-quality cells were obtained for subsequent analysis (Figure S2A and Table S1). Unsupervised clustering identified ten cell clusters that were consistently present across both wing types and species, yet showed distinct distribution patterns between FWD and HWD (Figure S2A).

We annotated these clusters into four major cell types based on marker genes (Figures 1C, 1D, and S2B–S2D; Tables S2 and S3): hemocytes (clusters 5, 6, and 10), marked by *Gata-beta* expression; tracheal cells (cluster 8), identified by *LOC101738228* (*Mipp1* ortholog); muscle cells (clusters 1, 2, and 9), characterized by *kettin* expression; and epithelial cells (clusters 3, 4, and 7), marked by *LOC101740813* (*elB* ortholog). Quantitative comparison revealed a striking difference in cellular composition between species: muscle cells were the predominant population in *B. mandarina* wing discs, whereas hemocytes and epithelial cells were significantly enriched in *B. mori* (Figures 1C and S2A; Table S1).

To spatially resolve these cell types, we performed transcriptomics using the 10× Visium platform (Figures S3A and S3B; and Tables S4 and S5). Integration with H&E staining and Robust cell-type deconvolution (RCTD) analysis localized muscle, tracheal, and epithelial cells primarily to the wing buds, while hemocytes were concentrated in associated hematopoietic organs (Figures 1E, S3C, and S3D). Together, these spatial analyses established a direct link between the spatial abundance of muscle cells within the wing bud and flight capability, implicating muscle cell development as a primary focus for understanding flight loss.

### Developmental trajectory analysis reveals arrested muscle differentiation in *B. mori*

To compare the developmental dynamics of flight organ cells between species, we reconstructed differentiation trajectories for hemocytes, epithelial cells, and muscle cells using RNA velocity, pseudotime, and partition-based graph abstraction (PAGA) analyses.

**Figure 2:**
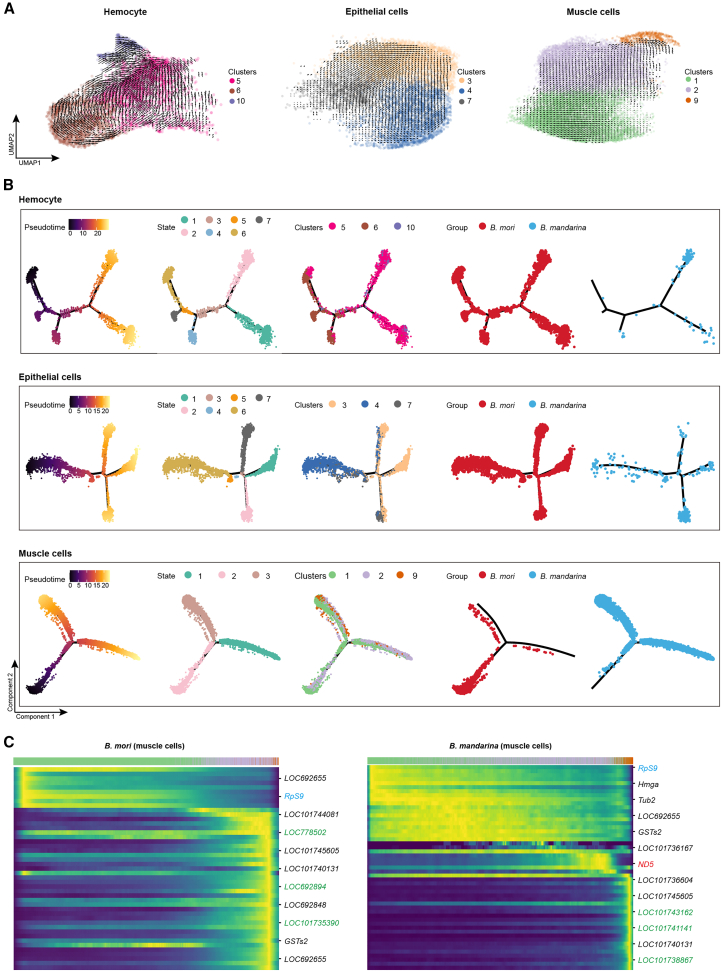
Developmental trajectories of wing disc cells in *B. mori* and *B. mandarina* (A) RNA velocity stream plots for hemocytes, epithelial cells, and muscle cells in *B. mori* and *B. mandarina*. (B) Pseudotime analysis of hemocytes, epithelial cells, and muscle cells in *B. mori* and *B. mandarina*. (C) Pseudotime analysis of muscle cell differentiation. Heatmap showing the expression dynamics of key genes along pseudotime in *B. mori* and *B. mandarina*. Genes are grouped and color-coded by functional category: ribosomal biogenesis (blue), mitochondrial energy metabolism (red), and flight muscle development (green). Expression levels are indicated by the color scale (high, yellow; low, and dark blue).

For hemocytes—significantly enriched in *B. mori*—developmental trajectories originated from cluster C6 (Figures 2A, S4A, and S4B). Pseudotime analysis revealed that *B. mori* hemocytes were distributed across multiple developmental states, whereas those of *B. mandarina* were predominantly concentrated in a single cell state (State 2) enriched for gene ontology terms related to transcriptional regulation (e.g., “negative regulation of gene expression”) (Figure 2B; Table S6). This indicates a marked divergence in hemocyte developmental landscapes between species.

Similarly, epithelial cell development, starting from cluster C4, showed distinct patterns between species (Figures 2A, S4A, and S4B). In *B. mandarina*, epithelial cells were predominantly located at the end of the trajectory, suggesting a well-defined terminal state. In contrast, *B. mori* epithelial cells were distributed across various states without resolving a clear endpoint, indicating an altered or incomplete differentiation program (Figure 2B and Table S6).

While these findings demonstrate that domestication impacted multiple cell lineages, the most profound and functionally decisive defect emerged within the muscle cell compartment. Muscle cells, which develop into adult flight muscles, exhibited a continuous differentiation trajectory from cluster C1 through C2 to C9 in *B. mandarina*, as supported by RNA velocity and PAGA analyses (Figures 2A–2C, S4A, and S4B). In contrast, this trajectory was severely truncated in *B. mori*, with muscle cells failing to resolve distinct terminal fates—a clear indication of developmental arrest.

Analysis of gene expression dynamics along the muscle cell pseudotime in *B. mandarina* revealed a coherent progression: ribosomal biogenesis genes (e.g., *Rps9*) were expressed early, followed by energy metabolism genes (e.g., *ND5*), and finally muscle developmental genes (e.g., *LOC101743162/Cht6*, *LOC101738867/pwn*). This logical, sequential activation was completely disrupted in *B. mori*, where the expression dynamics of these critical genes were uncoordinated along the pseudotime axis (Figure 2C). The expression trajectories of key genes, such as *ND5* and *pwn* exhibited marked differences between species, particularly during late development, reflecting a fundamental breakdown in the transcriptional program driving flight muscle maturation.

### Cell communication networks are rewired in flightless *B. mori*

**Figure 3:**
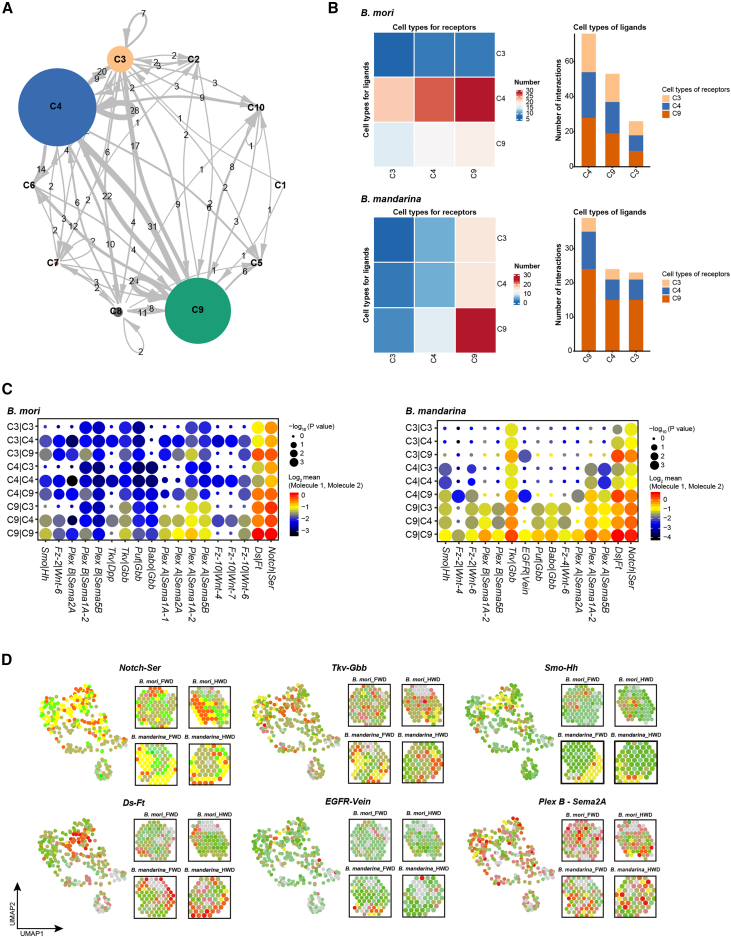
Cellular communication networks in silkworm flight organs (A) Circos plot depicting cell-cell interactions among ten cell clusters in wing discs, predicted by CellPhoneDB. Node size corresponds to the number of interactions; edge width represents the number of significant ligand-receptor pairs between clusters. (B) Heatmap and stacked bar charts showing the number and distribution of cell-cell interactions among clusters C3, C4, and C9 in *B. mori* and *B. mandarina*. (C) Dot plot of receptor-ligand pairs among clusters C3, C4, and C9 in *B. mori* (left) and *B. mandarina* (right). Dot size indicates the statistical significance of the interaction. Expression levels are color-coded (red, high; blue, low). (D) Spatial co-expression patterns of selected receptor-ligand pairs in forewing discs (FWD) and hindwing discs (HWD). Expression levels of receptor genes, ligand genes, and their co-expression are shown in red, green, and yellow, respectively.

To map cell-cell communication in silkworm wing discs, we analyzed ligand-receptor interactions using CellPhoneDB. This revealed that cluster C4 played a pivotal role in *B. mori*, engaging in strong interactions with clusters C3 and C9 (Figure 3A and Table S7). Comparative analysis identified six signaling pathways (Notch, Fat-Ds, Semaphorin, Wnt, BMP, and Hh) involving 18 receptor-ligand pairs in *B. mori*. In *B. mandarina*, the muscle cluster C9 served as the communication center, with seven conserved pathways (Notch, Fat-Ds, Semaphorin, Wnt, BMP, EGFR, and Hh) comprising 15 receptor-ligand pairs (Figures 3B and 3C).

Notably, we identified species-specific receptor-ligand interactions. Specific to *B. mori* were pairs in the Wnt (*Fz10-Wnt6*, *Fz10-Wnt7b*, and *Fz10-Wnt4*), Semaphorin (*PlexA-Sema1A*, *PlexB-Sema2A*), and BMP (*Tkv-Gbb*) pathways. In contrast, *Fz4-Wnt6*, *Fz2-Wnt4* (Wnt pathway) and *EGFR-Vein* (EGFR pathway) were unique to *B. mandarina*. Additionally, 12 receptor-ligand pairs—including *Notch-Ser* (Notch pathway), *Ds-Ft* (Ft-Ds pathway), and *Smo-Hh* (Hh pathway)—were common to both species but exhibited stronger communication intensity in *B. mandarina* (Figure 3C).

Spatial transcriptomics validated these interactions and mapped their expression domains. Pairs such as *Notch-Ser*, *Tkv-Gbb*, *PlexA*-*Sema1A*, and *PlexA*-*Sema2A* were co-expressed throughout the wing disc, while others (e.g., *Smo-Hh*, *EGFR-Vein*, and *Ds-Ft*) showed localized expression patterns (Figure 3D). Critically, pairs including *Notch*-*Ser*, *Tkv*-*Gbb*, *Smo*-*Hh*, *PlexA*-*Sema1A*, *PlexA*-*Sema2A*, and *PlexB*-*Sema1A* were enriched in wing bud regions, which also showed high expression of development-related genes (e.g., *Bmp-A*, *Fng*, and *Vg*) (Figures 3D and S5).

Within silkworm wing discs, the differential communication hubs, species-specific receptor-ligand repetoire, and attenuated interaction strengths in *B. mori* collectively suggest that its developmental program is not merely delayed but fundamentally rewired, likely contributing to flight deficiency.

### A hierarchical flight module is disrupted in *B. mori* flight muscles

**Figure 4:**
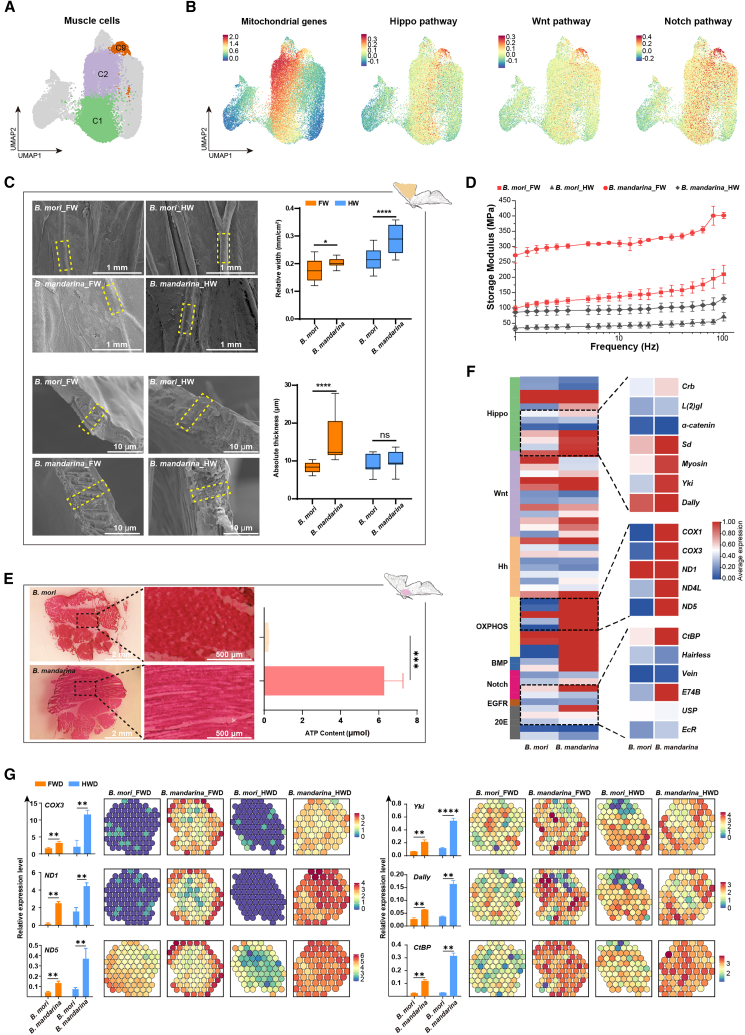
Disruption of the hierarchical flight module in *B. mori* flight muscles (A) UMAP plots of wing discs cells from *B. mori* and *B. mandarina*, with muscle cell clusters (C1, C2, and C9) highlighted. The proportion of muscle cells and the position of cluster C9 are indicated. (B) Normalized expression levels of mitochondrial genes and key components of the Hippo, Wnt, and Notch pathways across cells. (C) Left: scanning electron microscopy images of wing veins. Right: quantitative measurements of relative wing vein width and absolute thickness. Scale bars, 1 mm for wing width and 10 μm for wing thickness. (D) Comparison of the energy storage modulus (E′) of wings from *B. mori* and *B. mandarina*. (E) Left: microstructure of flight muscles. Right: ATP content in flight muscles. Scale bars, 2 mm for flight muscles and 500 μm for enlarged images. (F) Heatmap of hierarchical clustering showing the expression of representative genes from key signaling pathways in cluster C9 of *B. mori* and *B. mandarina wing disc cells*. (G) Validation of key gene expression. Left: RT-qPCR analysis of mRNA levels. Right: Spatial expression patterns from 10× Visium data. Data are presented as mean ± SD (*n* ≥ 3). ns, non-significant; ∗, *p* < 0.05; ∗∗, *p* < 0.01; ∗∗∗, *p* < 0.001; ∗∗∗∗, *p* < 0.0001.

Building on the identification of muscle cells as crucial for flight, we focused on cluster C9, which served as a communication hub in *B. mandarina* but was significantly diminished in *B. mori* (Figure 4A). These muscle cells exhibited high expression of mitochondria-related genes and components of key developmental pathways, including Hippo, Notch, and Wnt (Figures 4B and S6).

Morphological comparisons revealed substantial differences between the two species: *B. mandarina* possessed significantly wider and thicker wing veins, more regularly arranged flight muscles, and higher ATP content compared to *B. mori* (Figures 4C–4E). Consistent with these phenotypic deficits, at the molecular level, energy metabolism genes (e.g., *ND1*, *ND4*, *ND5*, *COX1*, and *COX3*) and development regulators (e.g., *Dally*, *CtBP*, *Yki*, *Myosin*, and *α-catenin*) were highly expressed in *B. mandarina* muscle cells but markedly downregulated in *B. mori* (Figure 4F).

Experimental validation and spatial gene expression analysis confirmed significantly higher expression of these genes in *B. mandarina* (Figures 4G and S7). Notably, several energy-related genes (*COX3*, *ND1*, and *ND4L*) and development regulators (*CtBP*, *myosin*, and *Sd*) were almost exclusively detected in *B. mandarina* wing discs, underscoring their essential roles in flight organ development.

Together, these results define a three-tiered, hierarchical module whose disintegration underlies flight loss in *B. mori*: (1) mitochondrial dysfunction (*COX3/ND1* suppression), (2) wing vein patterning defects (*Dally/CtBP* dysregulation), and (3) aborted flight muscle specification (*Yki* inactivation).

### Functional validation establishes causality and reveals dosage sensitivity

**Figure 5:**
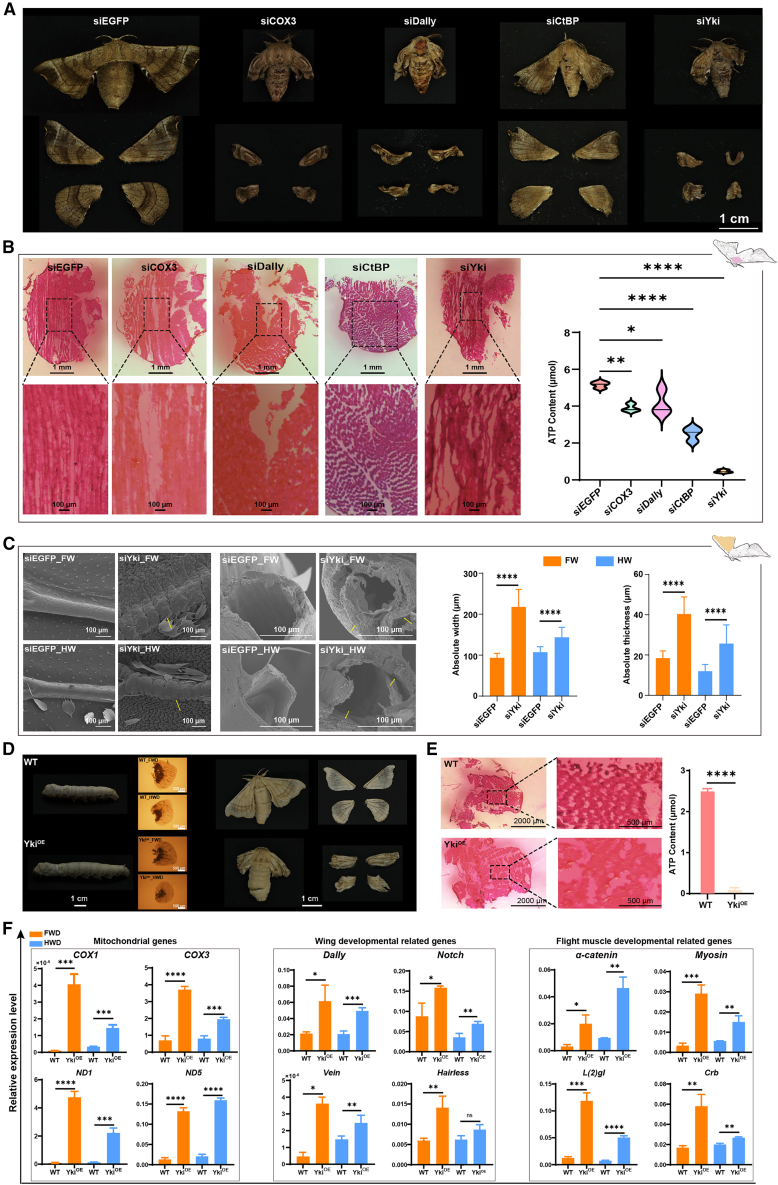
Functional validation of key genes reveals dosage sensitivity of the Hippo pathway (A) Adult wing phenotypes of *B. mandarina* following RNAi-mediated knockdown of *indicated genes in larvae*. siEGFP was used as a control. Scale bars, 1 cm. (B) Left: microstructure of *B. mandarina* flight muscles post-knockdown. Black boxes indicate areas shown in magnified views. Right: ATP content in flight muscles. Scale bars, 1 mm for flight muscles and 100 μm for enlarged images. (C) Left: scanning electron microscopy images of wing veins after *Yki* knockdown. Right: measurements of wing vein width and thickness. Yellow arrows highlight regions of vein widening and thickening. Scale bars, 100 μm. (D) Top: microscopic images of wing discs from day 6 fifth-instar (L5D6) Yki^OE^ larvae. Bottom: adult wing phenotypes of Yki^OE^ and wild-type controls. Scale bars, 1 cm for larvae and adults, 500 μm for wing discs. (E) Left: microstructure of flight muscles from L5D6 Yki^OE^ larvae. Black boxes indicate magnified areas. Right: ATP content in flight muscles. Scale bars, 2000 μm for flight muscles and 500 μm for enlarged images. (F) mRNA expression levels of key genes in wing discs of L5D6 Yki^OE^ larvae. Genes are grouped by function: mitochondrial, wing development, and flight muscle development. Data are presented as mean ± SD (*n* ≥ 3). ns, non-significant; ∗, *p* < 0.05; ∗∗, *p* < 0.01; ∗∗∗, *p* < 0.001; ∗∗∗∗, *p* < 0.0001.

To establish the causal role of the core genes within the flight module, we performed RNAi-mediated knockdown of *COX3*, *Dally*, *CtBP*, and *Yki* in *B. mandarina* wing discs (Figure S8A and Table S8). Strikingly, individual knockdown of each gene was sufficient to recapitulate aspects of the flightless phenotype. Knockdown of *COX3* caused dramatic reductions in adult wing size and ATP content, along with disorganized flight muscles (Figures 5A and 5B, Video S1). Similarly, knockdown of *Yki*, *Dally*, or *CtBP* also induced wing malformations and flight muscle disorganization, with *Yki* knockdown yielding the most severe phenotypes, underscoring its pivotal role (Figures 5A–5C).

Video S1. Verification of key candidate genes in *B. mandarina* and *H. armigera*

To further investigate *Yki* function, we generated a transgenic *B. mori* strain overexpressing *Yki* in wing discs (designated as Yki^OE^) (Figures S8B and S8C). Surprisingly, *Yki* overexpression exacerbated wing malformations, caused flight muscle loss, and reduced ATP content (Figures 5D, 5E, and S8D). Despite these adverse phenotypes, molecular analysis revealed that *Yki* overexpression significantly upregulated the expression of mitochondrial genes (e.g., *COX1*, *COX3*, *ND1*, and *ND5*), wing development genes (e.g., *Dally*, *Notch*, *Vein*, and *Hairless*), and flight muscle development genes (e.g., *α-catenin*, *Myosin*, *L(2)gl*, and *Crb*) (Figure 5F).

Collectively, these *in vivo* experiments demonstrate that dysregulation of the flight module genes is sufficient to impair flight organ function and cause flightlessness, while *Yki* overexpression also disrupts development, revealing a strict dosage requirement for Hippo signaling in flight muscle development.

### The flight module is essential and conserved in a *lepidopteran* pest

**Figure 6:**
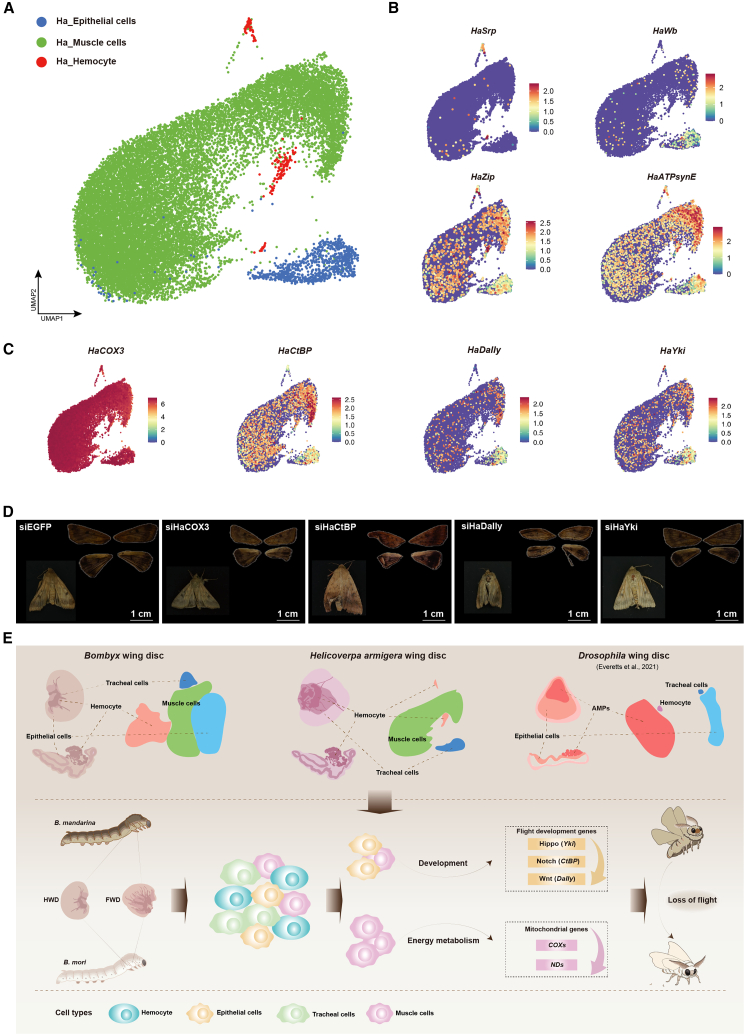
The flight module is essential and conserved in lepidopteran pest *H. armigera* (A) UMAP visualization of the annotated cell types in wing discs *from fifth-instar H. armigera* larvae. (B) Feature plots displaying the expression of canonical marker genes used for cell type annotation in (A). (C) UMAP plots visualizing the expression patterns of orthologs for key silkworm flight module genes in *H. armigera*. (D) Phenotypic consequences of RNAi-mediated knockdown in *H. armigera* wing discs. Compared to the *siEGFP* control, knockdown of indicated genes led to wing and flight muscle defects, resulting in flightlessness. Scale bars, 1 cm. (E) Comparative summary and proposed model. (Top) Single-cell atlas of wing discs from *B. mori*, *B. mandarina*, *H. armigera*, and *Drosophila melanogaster*, showing conserved cell type identities and validating the lepidopteran cell annotations. (Bottom) working model of flight loss during silkworm domestication, illustrating the hierarchical disintegration of the muscle-centric flight module involving mitochondrial dysfunction, patterning defects, and aborted muscle specification.

To evaluate the evolutionary conservation of the flight module, we generated a single-cell atlas of flight organs from the cotton pest *H. armigera* (Lepidoptera). From 18,718 high-quality cells (Tables S1 and S9), we identified three major cell types using canonical marker genes, including muscle cells (marked by *HaZip* and *HaATPsynE*), epithelial cells (marked by *HaWb*), and hemocytes (marked by *HaSrp*) (Figures 6A, 6B, and S9A–S9D; Tables S2 and S3).

Notably, *H. armigera* muscle cells were enriched for orthologs of the silkworm flight module genes, including mitochondrial genes (*HaCOXs* and *HaNDs*) and key developmental regulators (*HaYki*, *HaSd*, *HaDally*, *HaCtBP*, *Haα-catenin*, and *HaMyosin*) (Figure 6C). RNAi-mediated knockdown of these orthologs (*HaCOX3*, *HaDally*, *HaCtBP*, and *HaYki*) in *H. armigera* wing discs recapitulated key flightless phenotypes observed in silkworms, characterized by decreased ATP content, reduced wing area, disrupted flight muscle structure, and a complete loss of flight capacity (Figures 6D and S10A–S10D; Table S8; Video S1).

Collectively, these results demonstrate the functional conservation of the muscle-centric flight module across Lepidoptera, underscoring its potential as a target for precision pest control strategies designed to disrupt flight capacity.

## Discussion

The evolutionary loss of complex traits, such as flight, raises a fundamental question: does evolutionary regression proceed through random decay or organized rewiring? Our study in silkworms strongly supports the latter, demonstrating that the loss of flight resulted from the muscle-centered, hierarchical disintegration of an integrated developmental program.

The Hippo pathway effector *Yki*^20^^,^^21^ emerged as a critical, yet dosage-sensitive regulator with this program. In *B. mandarina*, *Yki* expression is maintained within an optimal range that supports the coordinated development of flight-capable organs. Accordingly, its knockdown severely compromises flight. In contrast, *B. mori* exhibits significantly reduced *Yki* expression. Crucially, overexpressing *Yki* in the domesticated background failed to restore flight and instead exacerbated wing defects and reduced ATP levels. This paradox indicates that the role of *Yki* cannot be understood in isolation; its function is embedded within a broader gene regulatory network. We propose that during domestication, the genetic context of the Hippo pathway—including upstream regulators, co-factors, and downstream targets—underwent coordinated evolutionary rewiring. The network stabilized in a new state compatible with rudimentary wing development but not with the high-energy demands of flight. In this altered context, simply elevating *Yki* dosage does not reconstitute the ancestral flight program but disrupts the evolved homeostasis, leading to deleterious outcomes. Thus, flight loss in *B. mori* is not caused by the simple downregulation of *Yki*, but by the collapse of the hierarchical, multi-tiered module in which Yki operates—a module whose integrity and optimal regulatory parameters were reshaped by domestication.

Our multi-omics approach reveals that the cellular and molecular divergence between domestic and wild silkworms has been established at the late larval stage, with key factors (e.g., *COX3*, *Yki*, *Dally*, and *CtBP*) already dysregulated. This positions the larval wing disc as a critical window for the initiation of flight loss. Future studies should determine the exact onset of this divergence and test the conservation of these mechanisms in other insects.

An unexpected finding was the significant expansion of hemocytes in *B. mori* flight organs. Given the known roles of hemocytes in wing morphogenesis and the presence of similar cells in *Drosophila* wing discs,^22^^,^^23^ this expansion may reflect a resource allocation trade-off under domestication. Energy previously devoted to maintaining costly flight structures may have been redirected toward hemocyte production or other functions, possibly enhancing adaptability of silkworms in controlled environments. This hypothesis, however, requires direct experimental validation.

The observed flight loss aligns with the classic evolutionary trade-off between flight and reproduction, known as the oogenesis-flight syndrome.^24^^,^^25^^,^^26^ Artificial selection for high fecundity and silk production in *B. mori* likely reinforced this trade-off, driving the downregulation of flight-related metabolic genes and reprogramming energy allocation toward reproductive traits. This organized physiological shift supports our model that flight degeneration is not a random decay but a selected adjustment of resource allocation strategies.

The conservation of this hierarchical module in the cotton pest *H. armigera* not only confirms its fundamental role in lepidopteran flight but also reveals a shared genetic vulnerability. This could be exploited for precision pest control by specifically impairing flight capacity—a trait essential for pest dispersal and reproduction^27^^,^^28^ —thereby offering a promising direction for developing species-specific pest management strategies without reliance on broad-spectrum toxins.

In summary, our work moves beyond descriptive accounts of trait loss to delineate the precise cellular and molecular steps of its regression. We establish that flight degeneration in silkworms occurred through the muscle-centered, coordinated collapse of a core genetic module and demonstrate its evolutionary conservation (Figure 6E). This model of hierarchical disintegration provides a new framework for understanding how complex traits degenerate evolutionarily, moving beyond the notion of mere decay. Furthermore, by identifying a conserved genetic architecture essential for flight, our work pinpoints a specific target for developing environmentally sustainable strategies to control *lepidopteran* pests.

### Limitations of the study

This study provides a detailed mechanistic model for flight loss in silkworms, yet several questions remain for future research. First, the upstream triggers and intermediary signals that link the three tiers of the hierarchy (e.g., how mitochondrial deficits lead to patterning gene downregulation) are unknown. Second, while we propose a resource reallocation hypothesis based on hemocyte expansion, direct evidence for a functional trade-off between flight and immunity is needed. Future studies could employ genetic or metabolomic approaches to simultaneously manipulate both pathways. Finally, our focus on the wing disc invites exploration of whether this hierarchical breakdown is a tissue-specific phenomenon or part of an organism-wide metabolic shift.

## Resource availability

### Lead contact

Further information and requests for resources and reagents should be directed to and will be fulfilled by the lead contact, Hanfu Xu (xuhf@swu.edu.cn).

### Materials availability

This study did not generate new unique reagents.

### Data and code availability

• Data: The single-cell and spatial transcriptomics data generated in this study have been deposited in the GEO database with the accession number GSE253665, GSE253666, and GSE253667. The raw sequencing data are available in the NCBI SRA under BioProject accessions PRJNA1066737, PRJNA1066738, and PRJNA1066739.
• Code: This paper does not report original code.
• Additional information: Any additional information required to reanalyze the data reported in this article is available from the lead contact upon request.

## Acknowledgments

This work was supported by grants from the 10.13039/501100001809National Natural Science Foundation of China (32272941), the Guangxi Science and Technology Program (AB241484024), the Fundamental Research Funds for the Central Universities (SWU-XJPY202309), and the Cocoon Silk Development Project of Chongqing Municipal Commission of Commerce (20240530100526500; 20250305152052389).

## Author contributions

R.L.: methodology, formal analysis, and writing - original draft. C.Z.: formal analysis and writing - original draft. J.H.: formal analysis and writing - original draft. Y.B.: methodology, formal analysis, and data curation. Y.R.: formal analysis and validation. Y.M.: formal analysis and validation. Y.T.: resources and validation. Y.M.: methodology and funding acquisition. Z.Z.: methodology. K.G.: methodology. K.D.: resources. X.L.: resources. Y.Z.: resources. W.T.: resources. H.X.: funding acquisition, conceptualization, supervision, and writing - review and editing.

## Declaration of interests

The authors declare no competing interests.

## STAR★Methods

### Key resources table

**Table:** 

REAGENT or RESOURCE	SOURCE	IDENTIFIER
**Biological samples**		

FWDs and HWDs of *Bombyx mori* in L5D6 larvae were used for scRNA-seq, stRNA-seq, real-time qPCR. FWs and HWs of *Bombyx mori* in adults within 24 h after emergence were used for the morphological observation and the determination of elastic modulus.	The State Key Laboratory of Resource Insects (Southwest University, China)	N/A
FWDs and HWDs of Yki^OE^ in L5D6 larvae were used for the morphological observation and real-time qPCR. FWs and HWs of Yki^OE^ in adults within 24 h after emergence were used for the morphological observation.	The State Key Laboratory of Resource Insects (Southwest University, China)	N/A
FWDs and HWDs of *Bombyx mandarina* in L5D6 larvae were used for scRNA-seq, stRNA-seq, real-time qPCR. FWs and HWs of *Bombyx mandarina* in adults within 24 h after emergence were used for the morphological observation and the determination of elastic modulus.	Collected from Anhui Province of China	N/A
FWDs and HWDs of *Helicoverpa armigera* in L5D3 larvae were used for scRNA-seq and real-time qPCR. FWs and HWs of *Helicoverpa armigera* in adults within 24 h after emergence were used for the morphological observation.	Collected from Henan Province of China	N/A

**Critical commercial assays**		

Chromium Next GEM Single Cell 3ʹ Kit v3.1	10× genomics	Cat#1000268
RNase inhibitors	Sigma	Cat#3335399001
Visium Spatial Gene Expression Slide & Reagent Kit	10× genomics	Cat#1000184
PrimeScript™ RT Reagent Kit	Takara	Cat#RR047A
TB Green® Premix Ex Taq^TM^ Ⅱ	Takara	Cat# RR820A
E.Z.N.A. MicroElute Total RNA Kit	Omega Biotek	Cat#R6831-01

**Deposited data**		

Reference genome of *Bombyx mori* and *Bombyx mandarina*	NCBI	https://ftp.ncbi.nlm.nih.gov/genomes/all/GCF/014/905/235
Reference genome of *H. armigera*	NCBI	https://ftp.ncbi.nlm.nih.gov/genomes/all/GCF/023/701/775/G
scRNA-seq datasets of *Bombyx mori* and *Bombyx mandarina*	This paper	GEO: GSE253666 https://www.ncbi.nlm.nih.gov/geo/query/acc.cgi?acc=GSE253666 BioProject: PRJNA1066737 https://www.ncbi.nlm.nih.gov/bioproject/?term=PRJNA1066737
scRNA-seq datasets of *Helicoverpa armigera*	This paper	GEO: GSE253667 https://www.ncbi.nlm.nih.gov/geo/query/acc.cgi?acc=GSE253667 BioProject: PRJNA1066738 https://www.ncbi.nlm.nih.gov/bioproject/?term=PRJNA1066738
Spatial transcriptomic datasets	This paper	GEO: GSE253665 https://www.ncbi.nlm.nih.gov/geo/query/acc.cgi?acc=GSE253665 BioProject: PRJNA1066739 https://www.ncbi.nlm.nih.gov/bioproject/?term=PRJNA1066739

**Experimental models: Organisms/strains**		

Silkworm/*Bombyx mori*	The State Key Laboratory of Resource Insects (Southwest University, China)	N/A
Silkworm/*Bombyx mandarina*	Collected from Anhui Province of China	N/A
cotton bollworm/*Helicoverpa armigera*	Collected from Henan Province of China	N/A

**Oligonucleotides**		

Primer sequences used for real-time qPCR, see Table S8	This paper	N/A
siRNA sequences used for the candidate genes, see Table S8	This paper	N/A

**Software and algorithms**		

Cell Ranger v7.0.1	Butler et al.^29^	https://www.10xgenomics.com/support/software/cell-ranger/latest/release-notes/cr-release-notes
DoubletFinder package v2.0.2	McGinnis et al.^30^	https://github.com/chris-mcginnis-ucsf/DoubletFinder
Seurat v4.0.0	Butler et al.^29^	https://github.com/satijalab/seurat
R package batchelor v1.6.3	Haghverdi et al.^31^	https://bioconductor.org/packages/release/bioc/vignettes/scran/inst/doc/scran.R
the Python script velocyto.py	Svensson et al.^32^	https://github.com/velocyto-team/velocyto.py
scVelo	Bergen et al.^33^	https://scvelo.readthedocs.io/
CellPhoneDB v3.1	Efremova et al.^34^	https://github.com/ventolab/CellphoneDB
RCTD v1.2.0	Cable et al.^35^	https://github.com/dmcable/RCTD

### Experimental model and study participant details

The domestic silkworm *Bombyx mori* (*Dazao* strain) was reared under standard laboratory conditions at Southwest University on fresh mulberry leaves at 25–28°C. The wild silkworm *Bombyx mandarina* was collected from Anhui Province, China. The cotton bollworm *Helicoverpa armigera* was collected from Henan Province, China. For all species, larvae were staged precisely at the designated days of the fifth instar (L5D6 for *Bombyx* species, L5D3 for *H. armigera*) for tissue dissection, as these stages represent a key phase of active development for the wing discs prior to wandering. All experimental procedures complied with ethical guidelines at Southwest University.

### Method details

#### Single-cell RNA sequencing

##### Sample preparation and library construction

Forewing discs (FWD) and hindwing discs (HWD) were dissected from staged larvae. Tissues were pooled by species and wing type (*B. mori*: 72 FWD, 71 HWD; *B. mandarina*: 24 FWD, 24 HWD; *H. armigera*: 48 FWD, 48 HWD). Pooled tissues were dissociated in PBS with 0.04% BSA to generate single-cell suspensions. Cell viability was confirmed to be >80% before proceeding. Libraries were constructed using the Chromium Single Cell 3′ Kit (10x Genomics) and sequenced on an Illumina NovaSeq platform.

#### Spatial transcriptomics

##### Tissue processing and sequencing

Freshly dissected FWD and HWD from *B. mori* and *B. mandarina* L5D6 larvae were embedded in OCT compound and snap-frozen. Tissues were cryosectioned at 10 μm thickness and mounted onto Visium Spatial Gene Expression slides (10x Genomics). Sections were stained with H&E and imaged using a 3D HISTECH Pannoramic MIDI FL slide scanner. Spatially barcoded cDNA libraries were constructed following the Visium protocol and sequenced on an Illumina NovaSeq platform.

#### Morphological and functional phenotyping

##### Wing and muscle morphology

Adult wings from *B. mori*, *B. mandarina*, and *H. armigera* moths (within 24 h post-eclosion) were imaged. Wing area and vein morphology were quantified using ImageJ (v1.47). For flight muscle analysis, thoraxes were dissected, embedded in OCT, cryosectioned at 10-25 μm, stained with H&E, and imaged using an Olympus DP80 microscope.

##### Scanning electron microscopy (SEM)

Adult wings were sputter-coated with gold and imaged using a Hitachi SU3500 SEM.

##### ATP content measurement

Flight muscles (20 mg) from newly eclosed moths were homogenized, and ATP levels in the supernatant were quantified using an Enhanced ATP Assay Kit (Beyotime). Luminescence was measured on a Gen5 Microplate Reader (BioTek), and concentrations were calculated against a standard curve.

##### Dynamic mechanical analysis (DMA)

The elastic modulus (E′) of adult wings was measured using a dynamic mechanical analyzer (TA Instruments). Wing strips (1 × 0.5 cm) were subjected to a frequency sweep from 1 to 100 Hz at 0.1% strain.

##### Flight ability assay

Flight capacity of individual moths was assessed within 24 h of eclosion in a controlled arena. Flight behavior was recorded using a digital video camera (Sony) and analyzed manually.

#### Molecular analyses

##### Quantitative real-time PCR (qRT-PCR)

Total RNA was extracted from wing discs using the E.Z.N.A. MicroElute Total RNA Kit (Omega Bio-tek). cDNA was synthesized using the PrimeScript RT Reagent Kit (Takara). qRT-PCR was performed on a QTOWER 2.0 system (Analytik Jena) using SYBR Premix Ex Taq (Takara). Each 20 μL reaction contained 2 μL cDNA, 0.8 μL of each primer, 10 μL SYBR Premix, 0.4 μL ROX Reference Dye, and 6 μL ddH_2_O. Thermal cycling conditions were: 95°C for 3 s, followed by 40 cycles of 95°C for 3 s and 60°C for 30 s. Gene expression was normalized to an internal reference gene, and the 2ˆ(-ΔΔCt) method was used for relative quantification. Three biological replicates were analyzed per group. All primer sequences are listed in Table S8.

##### RNA interference (RNAi)

Gene-specific siRNAs were designed using siDirect2.0 website and synthesized commercially (Sangon Biotech). A total of 5 μL of siRNA (1 μg/μL) or control siEGFP was microinjected into the second thoracic segment of *B. mandarina* (L5D5) or *H. armigera* (L5D2) larvae (n=30 per group). Tissues were collected 24 h post-injection for molecular analysis, and the remaining insects were reared to adulthood for phenotypic assessment.

##### Transgenic silkworm generation

Transgenic silkworms overexpressing *Yki* in the wing disc (Yki^OE^) were generated by crossing a UAS-Yki strain (UYki) with an A4IP-GAL4 driver strain, which exhibits high GAL4 expression in the wing disc.^36^ The F1 progeny (Yki^OE^) and wild-type controls were reared on mulberry leaves at 26°C–28°C.

### Quantification and statistical analysis

#### Single-cell and spatial transcriptomics data analysis

Raw sequencing data were processed using Cell Ranger (v7.0.1)^29^ for alignment to the respective reference genomes (*B. mori* and *B. mandarina*: Bmori_2016v1.0; *H. armigera*: HaSCD2). Downstream analysis was performed in Seurat (v4.0.0).^29^ Low-quality cells were filtered out (genes <100 or >4000; UMIs <1000), and doublets were removed using DoubletFinder (v2.0.2).^30^ Data integration was performed using the batchelor package (v1.6.3).^31^ Cell clusters were visualized using UMAP. Cell types were annotated based on canonical marker genes. Differential expression analysis were performed using the FindMarkers function (presto algorithm) with thresholds of |log_2_(fold change)| > 0.58 and adjusted P-value < 0.05. Developmental trajectories were inferred with Monocle3. RNA velocity analysis was performed by counting spliced/unspliced reads with velocyto.py^32^ and modeling with scVelo.^33^ Cell-cell communication was analyzed using CellPhoneDB (v3.1).^34^ Spatial transcriptomics data were processed using Space Ranger (v1.2.0) and analyzed in Seurat. Cell-type deconvolution was performed using RCTD (v1.2.0).^35^

#### Statistical analysis

All statistical analyses were performed using GraphPad Prism 9.0. Data are presented as mean ± standard deviation (s.d.). Differences between two groups were assessed by two-tailed unpaired Student’s t-test. Multiple group comparisons were performed by one-way ANOVA followed by an appropriate post-hoc test. A P-value of < 0.05 was considered statistically significant (∗, *p* < 0.05; ∗∗, *p* < 0.01; ∗∗∗, *p* < 0.001; ∗∗∗∗, *p* < 0.0001). Sample sizes (n) represent biological replicates and are specified in the figure legends.

## Supplemental information

Document S1. Figures S1–S10

Table S1. Cell statitics before and after QC and number of cells in each cluster in scRNA-seq data of *B. mori*, *B. mandarina* and *H. armigera*, related to Figures 1 and 6

Table S2. Marker genes for each cluster in scRNA-seq data of *B. mori*, *B. mandarina* and *H. armigera*, related to Figures 1 and 6

Table S3. Top 20 of GO and KEGG enrichment analysis for each cluster in scRNA-seq data of *B. mori*, *B. mandarina* and *H. armigera*, related to Figures S2 and S9

Table S4. Marker genes for each cluster of stRNA-seq data in flight organs of *B. mori* and *B. mandarina*, related to Figure 1

Table S5. Top 20 of GO and KEGG enrichment analysis for each cluster of stRNA-seq in *B. mori* and *B. mandarina*, related to Figure S3

Table S6. GO enrichment analysis of the top 30 differential genes in each pseudotime state of *B. mori* and *B. mandarina*, related to Figure 2

Table S7. Information of receptor-ligands detected in flight organs of *B. mori* and *B. mandarina*, related to Figure 3

Table S8. Primer and siRNA sequences used for the analysis of candidate genes, related to Figures 4, 5, S7, S8, and S10

Table S9. Standard detection of cell samples from *Helicoverpa armigera* samples, related to Figure 6

Data S1. Source data, related to Figures 4 and 5

Data S2. Source data, related to Figures S1, S7, S8, and S10
