# Supplementary material for: A muscle-centered hierarchical breakdown underlies flight loss during silkworm domestication
Source: iScience. 2026 Jan 29;29(2):114846. doi: 10.1016/j.isci.2026.114846 (PMC12915254; doi:10.1016/j.isci.2026.114846)
Supplement: Document S1. Figures S1–S10 [file mmc1.pdf]

iScience, Volume *29*

Supplemental information

A muscle-centered hierarchical breakdown underlies flight loss during silkworm domestication

Rongpeng Liu, Chen Zhao, Jie Hu, Yongbing Ba, Yiting Ran, Yuanyuan Mu, Yiyun Tang, Yan Ma, Zhiming Zhang, Kaiqi Guo, Keshu Dong, Xiao Li, Yumeng Zhu, Wei Tan, and Hanfu Xu

Supplementary Figures

Supplementary Figure 1

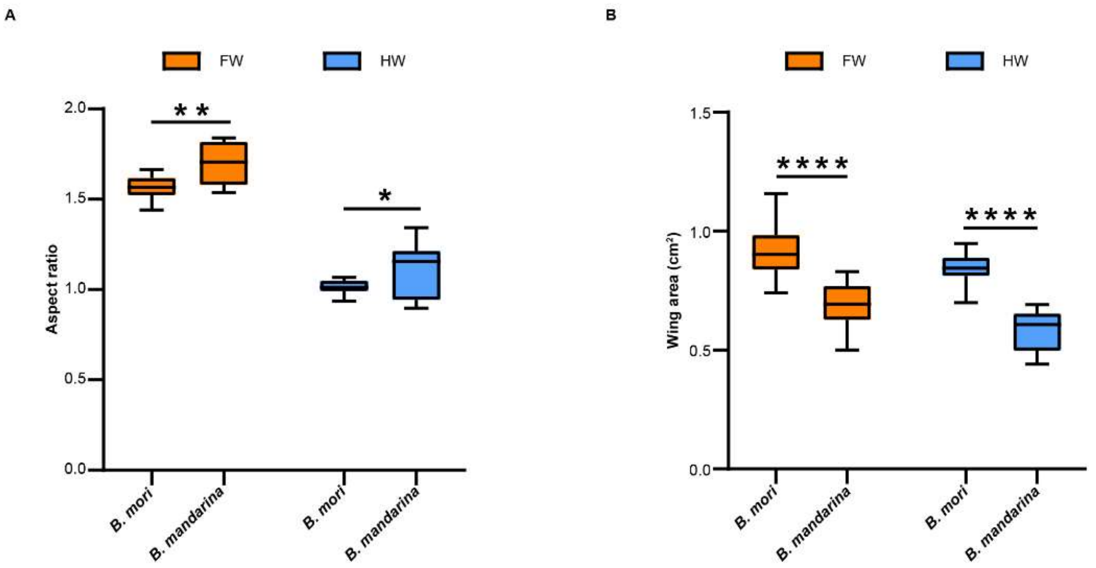

Supplementary Figure 1: Morphological differences between the wings of *B. mori* and *B. mandarina*

(A) Aspect ratio of the forewing (FW) and hindwing (HW) in *B. mori* and *B. mandarina* (n = 12). The aspect ratio (long axis/short axis, as defined by yellow and green lines in Figure 1) indicates wing shape, with higher values corresponding to a more elongated shape.

(B) Wing area of the FW and HW in *B. mori* (n = 30) and *B. mandarina* (n = 16). Data are presented as mean ±SD. ns, non-significant; *, $p < 0.05$; **, $p < 0.01$; ***, $p < 0.001$; ****, $p < 0.0001$.

Supplementary Figure 2

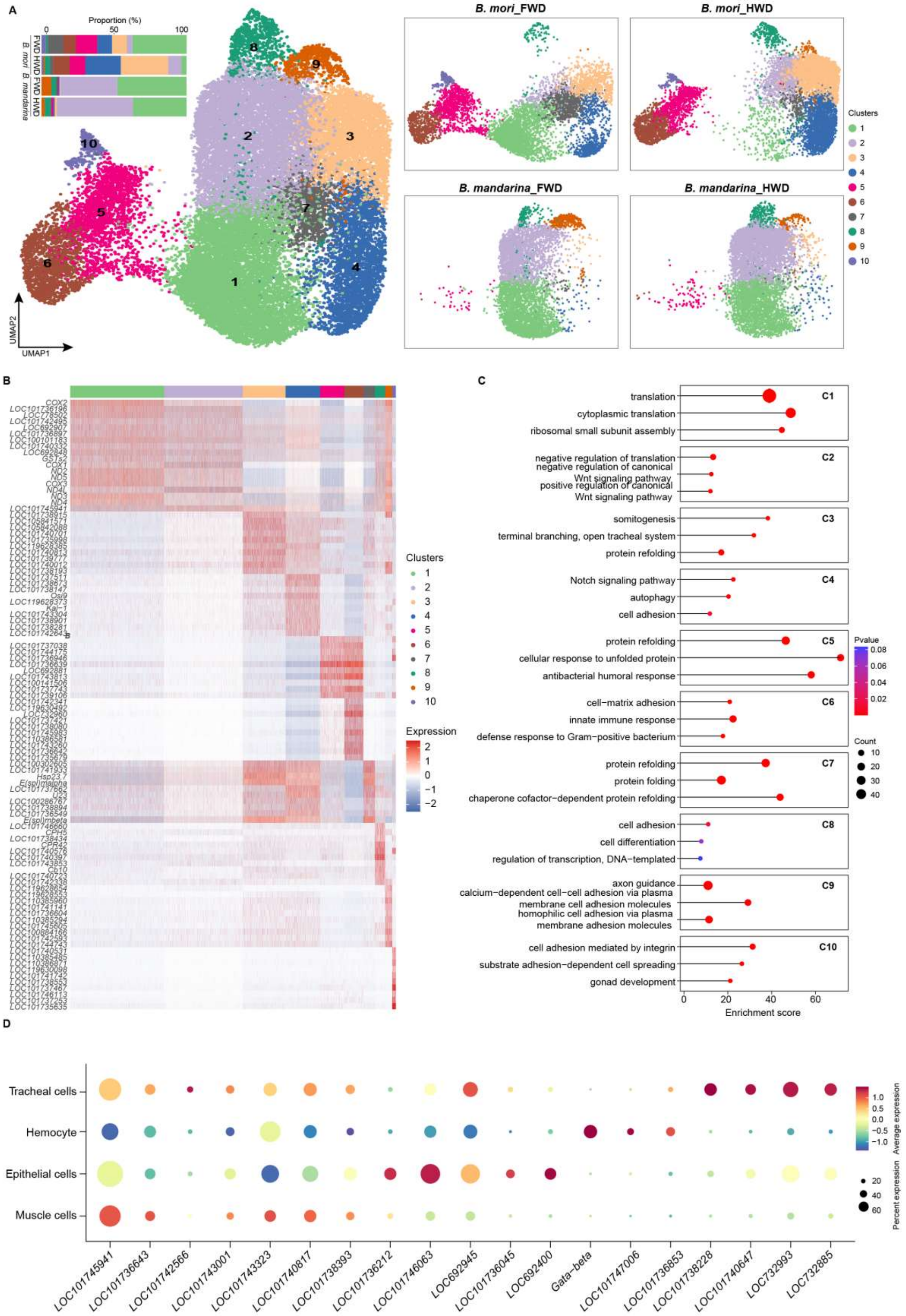

Supplementary Figure 2: Single-cell atlas of wing discs from *B. mori* and *B. mandarina*

(A) UMAP visualization of cell clusters and proportion abundance of each cell cluster from four wing discs samples: *B. mori*_FWD, *B. mori*_HWD, *B. mandarina*_FWD, and *B. mandarina*_HWD.

(B) Heatmap of the top 10 markers for each cluster.

(C) GO enrichment analysis of each cluster.

(D) Dot plot showing the expression levels of signature genes for the four annotated cell types.

(UMAP: uniform manifold approximation and projection; FWD: forewing disc; HWD: hindwing disc)

Supplementary Figure 3

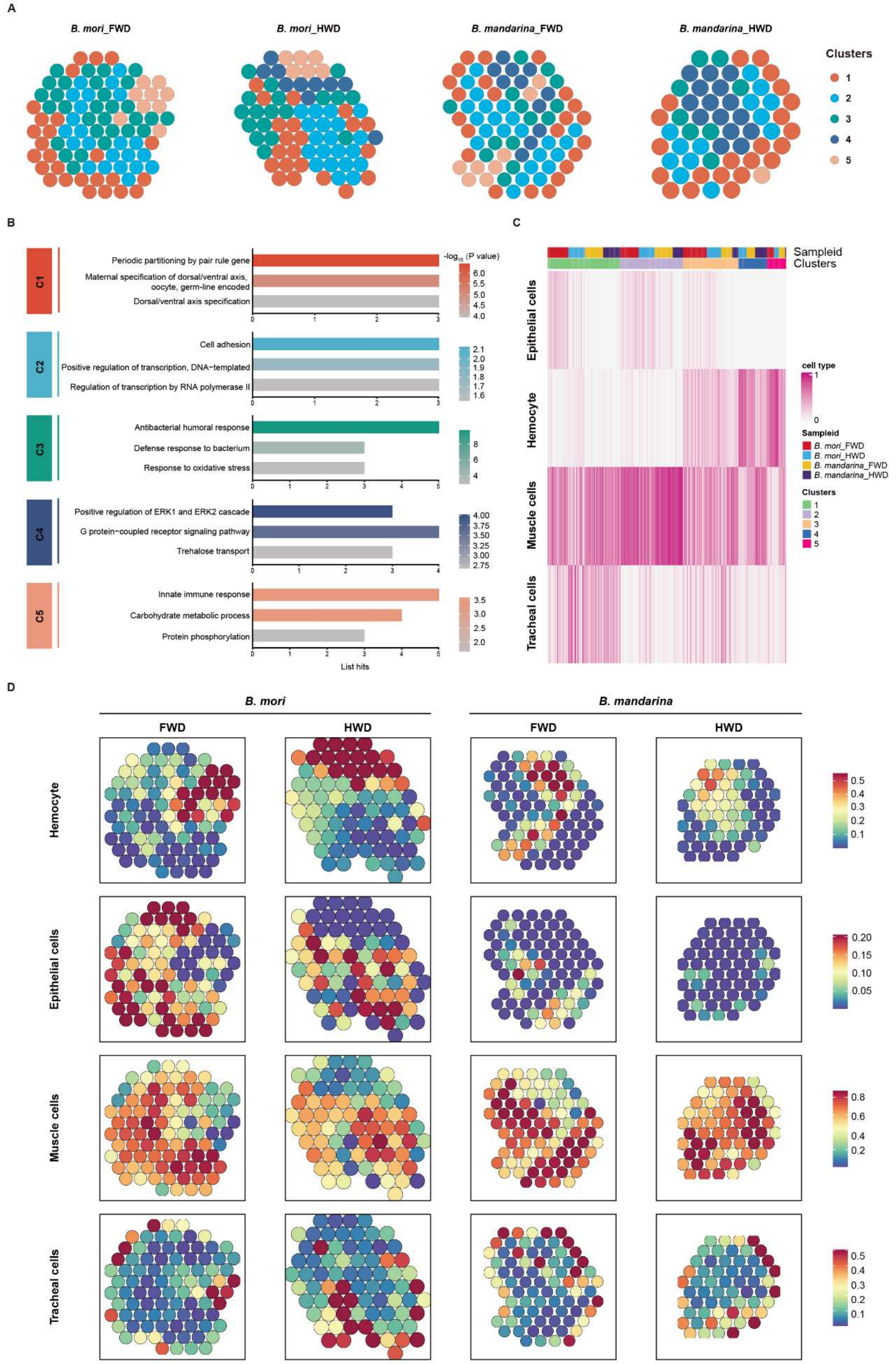

Supplementary Figure 3: Spatial cellular architecture of wing discs from *B. mori* and *B. mandarina*

(A) Spatial transcriptome atlas of *B. mori* and *B. mandarina* wing discs. Colors represent distinct cell clusters.

(B) Gene Ontology (GO) functional enrichment analysis for cell clusters defined by spatial transcriptomics (ST). Color intensity represents the enrichment score.

(C) Heatmap showing the deconvoluted proportions of scRNA-seq-defined cell types within ST spots. Darker pink indicates a higher cell type proportion.

(D) Spatial mapping of the four major cell types from scRNA-seq data onto the ST atlas.

Supplementary Figure 4

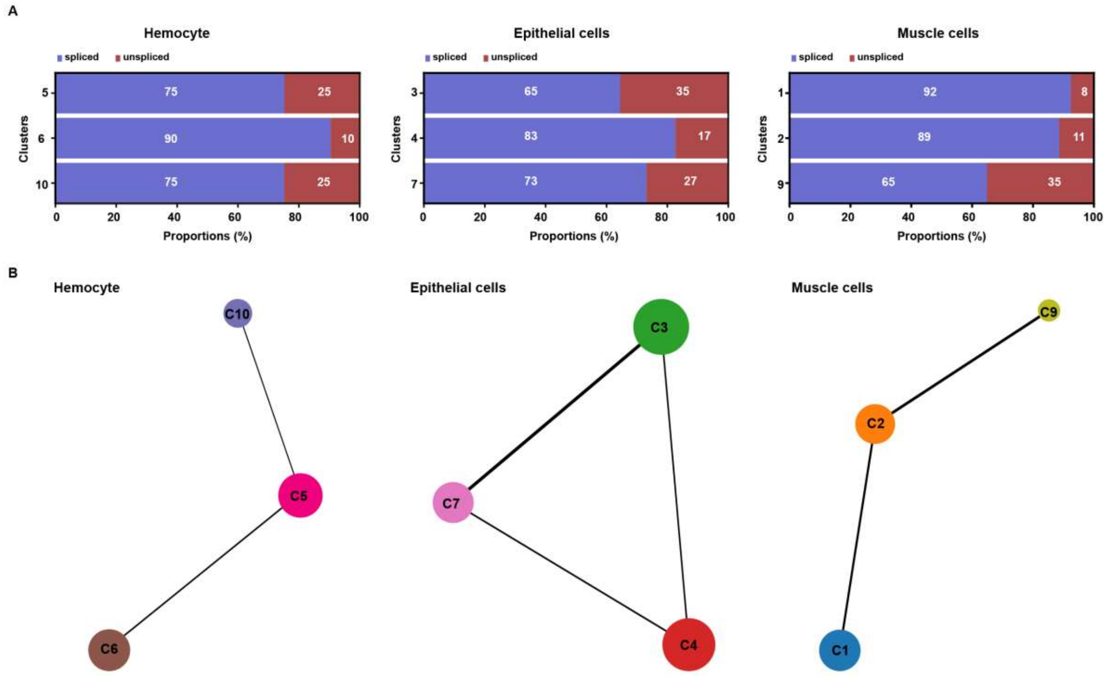

Supplementary Figure 4: Developmental trajectory of cell types in wing discs from *B. mori* and *B. mandarina*

(A) Proportion of spliced versus unspliced mRNAs for hemocytes, epithelial cells, and muscle cells. The ratio is used to infer RNA velocity and developmental direction.

(B) Partition-based graph abstraction (PAGA) plot illustrating the potential developmental connections between cell clusters. Edge thickness indicates the confidence of the connection.

Supplementary Figure 5

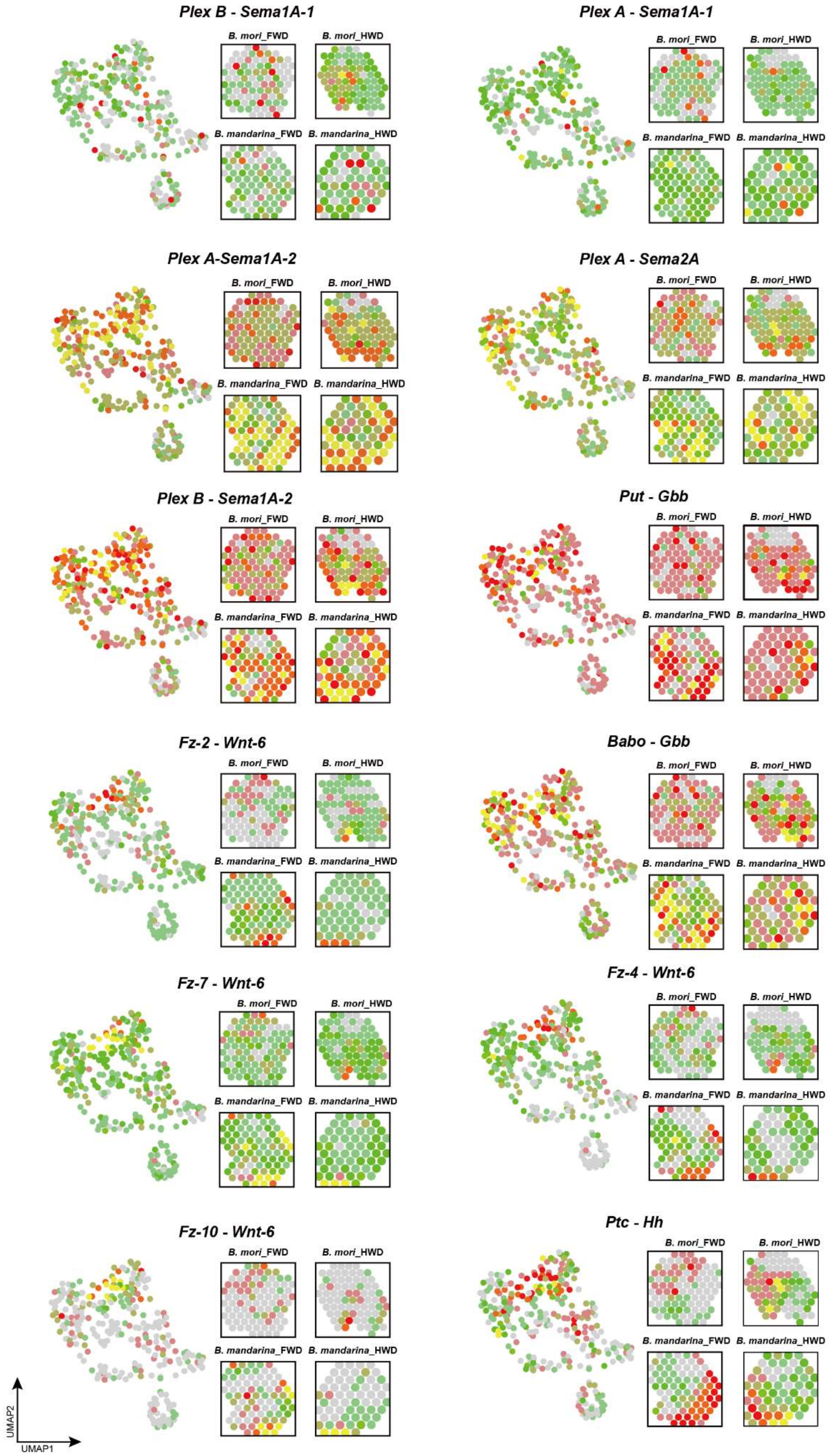

Supplementary Figure 5: Spatial co-expression patterns of receptor–ligand pairs

Spatial expression patterns of representative receptor–ligand pairs in *B. mori* and *B. mandarina* FWD and HWD. For each pair, the UMAP plot (left) shows its distribution in the spatial transcriptome atlas, and the image on the right shows the spatial mapping derived from single-cell data. (FWD: forewing disc; HWD: hindwing disc)

Supplementary Figure 6

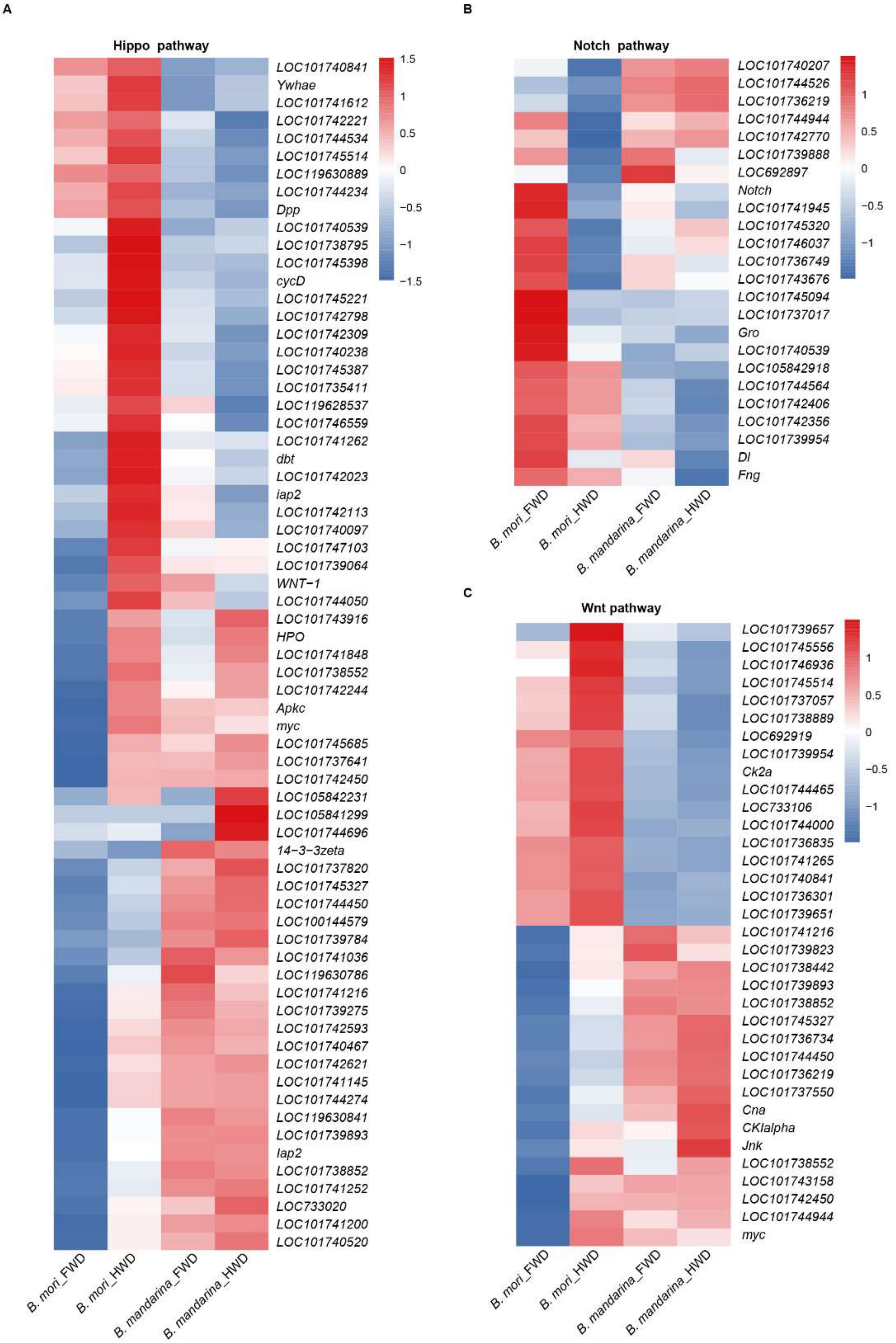

Supplementary Figure 6: Expression of Hippo, Notch, and Wnt pathway genes in wing discs

Heatmap showing the expression of genes in the Hippo (A), Notch (B), and Wnt (C) signaling pathways in the *B. mori*_FWD, *B. mori*_HWD, *B. mandarina*_FWD, and *B. mandarina*_HWD samples. Colors from red to blue represent gene expression levels from high to low, respectively. (FWD: forewing disc; HWD: hindwing disc)

Supplementary Figure 7

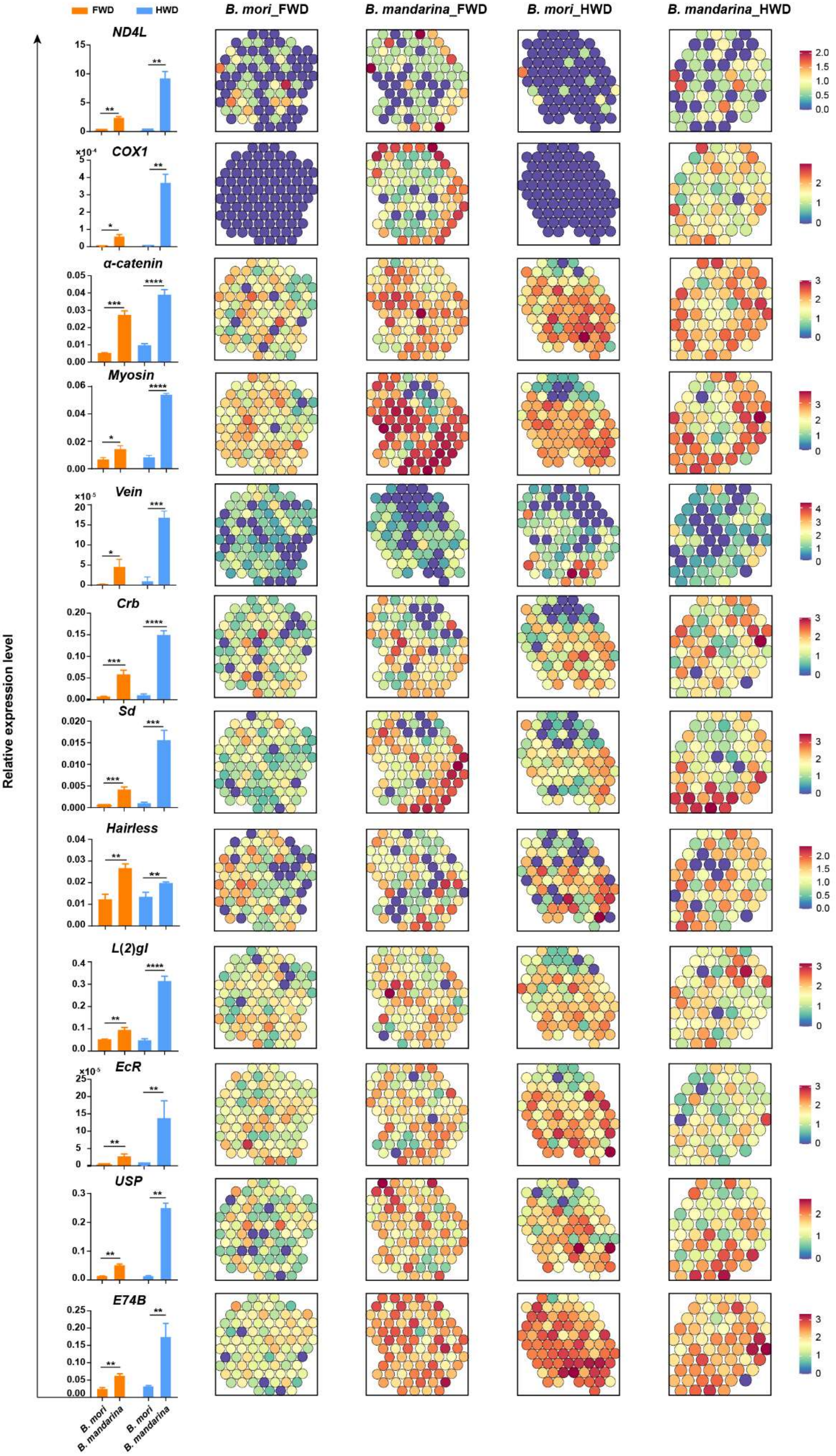

Supplementary Figure 7: Expression and spatial distribution of representative marker genes in wing discs from *B. mori* and *B. mandarina*

mRNA expression levels (left) and spatial distribution (right) of representative marker genes in *B. mori* and *B. mandarina* forewing disc (FWD) and hindwing disc (HWD). Data are presented as mean ±SD (n = 3). ns, non-significant; *, $p < 0.05$; **, $p < 0.01$; ***, $p < 0.001$; ****, $p < 0.0001$.

Supplementary Figure 8

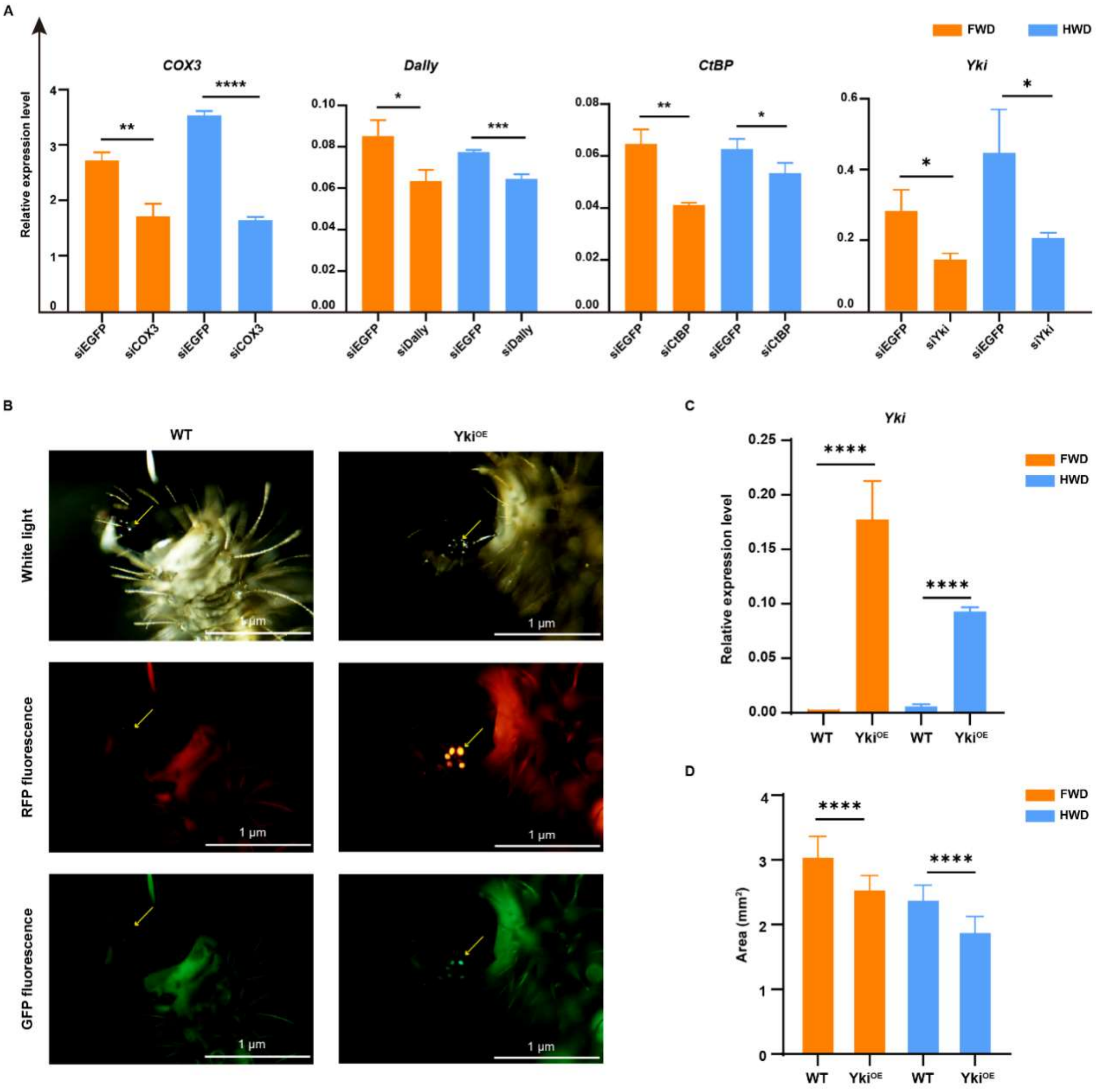

Supplementary Figure 8: Validation of key genes associated with the flight capacity of silkworms

(A) qRT–PCR validation of *COX3*, *Dally*, *CtBP*, and *Yki* mRNA levels following RNAi knockdown in *B. mandarina* wing discs.

(B) Generation of *Yki* overexpression transgenic silkworms (Yki^{OE}). Adult moths exhibiting RFP and GFP fluorescence in the compound eye (yellow arrow). Wild-type (WT) served as the control. Scale bars, 1μm

(C) *Yki* mRNA levels in L5D6 wing discs of Yki^{OE} strains.

(D) Area of L5D6 wing discs in Yki^{OE} strains. Data are presented as mean ±SD ($n \geq 3$). ns, non-significant; *, $p < 0.05$; **, $p < 0.01$; ***, $p < 0.001$; ****, $p < 0.0001$. (L5D6: day 6 fifth- instar; FWD: forewing disc; HWD: hindwing disc)

Supplementary Figure 9

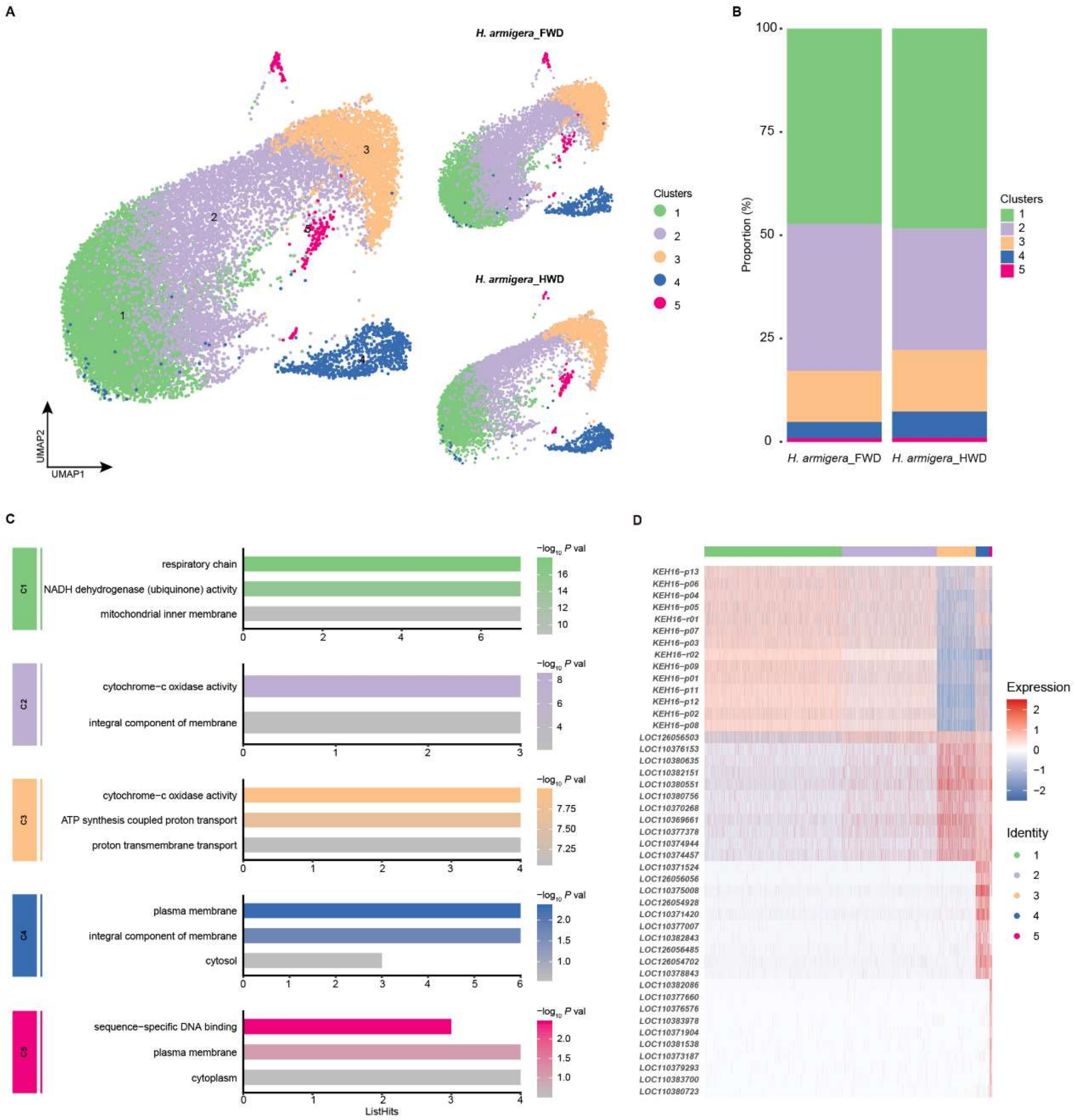

Supplementary Figure 9: Single-cell transcriptome atlas of wing discs from *Helicoverpa armigera*

(A) UMAP visualization of cell clusters from *H. armigera* FWD and HWD samples.

(B) Proportional abundance of cell clusters from *H. armigera* FWD and HWD samples.

(C) GO functional enrichment analysis for the five cell clusters. Color intensity represents the enrichment score.

(D) Heatmap of the top 10 marker genes for the five cell clusters.

(UMAP: uniform manifold approximation and projection; FWD: forewing disc; HWD: hindwing disc).

Supplementary Figure 10

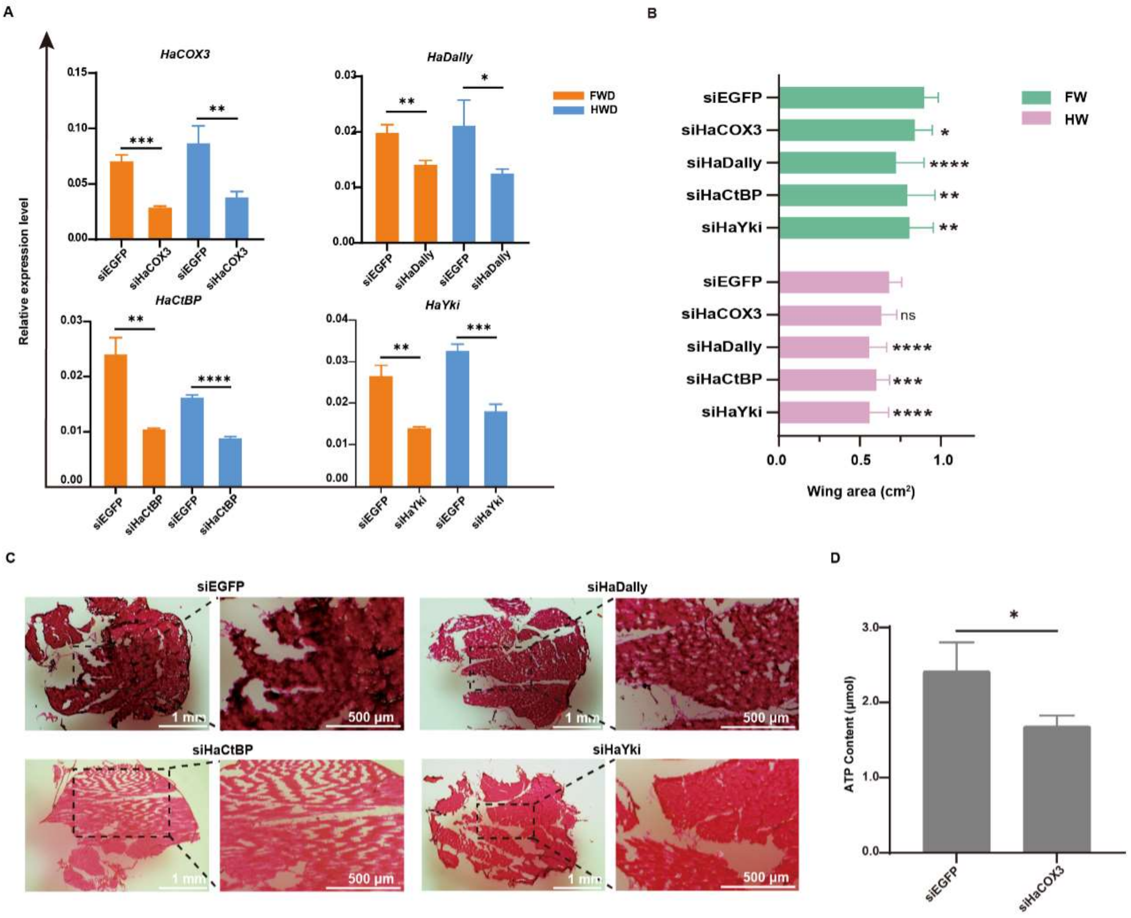

Supplementary Figure 10: Functional validation of key genes in *Helicoverpa armigera*

(A) qRT-PCR analysis of *HaCOX3*, *HaDally*, *HaCtBP*, and *HaYki* mRNA levels after RNAi knockdown in *H. armigera* wing discs.

(B) Wing area following RNAi knockdown of the target genes.

(C) Flight muscle microstructure after RNAi knockdown. The black box indicates the region shown in the magnified view. Scale bars, 1 mm for flight muscles and 500 μm for enlarged images.

(D) ATP content in flight muscle after *HaCOX3* knockdown. Data are presented as mean ±SD ($n \geq 3$). ns, non-significant; *, $p < 0.05$; **, $p < 0.01$; ***, $p < 0.001$; ****, $p < 0.0001$. (FWD: forewing disc; HWD: hindwing disc; FW: forewing; HW: hindwing)
